# First steps towards international competency goals for residency training: a qualitative comparison of 3 regional standards in anesthesiology

**DOI:** 10.1186/s12909-021-03007-w

**Published:** 2021-11-10

**Authors:** Clément Buléon, Reuben Eng, Jenny W. Rudolph, Rebecca D. Minehart

**Affiliations:** 1 Department of Anesthesiology, Intensive Care and Perioperative Medicine, Caen Normandy University Hospital, 6th Floor, Caen, France; 2 Medical School, University of Caen Normandy, Caen, France; 3 Center for Medical Simulation, Boston, MA USA; 4 Department of Anesthesia, Rockyview General Hospital, Calgary, AB Canada; 5 Department of Anesthesiology, Perioperative and Pain Medicine, University of Calgary, Calgary, AB Canada; 6 Department of Anesthesia, Critical Care and Pain Medicine, Massachusetts General Hospital, Boston, MA USA; 7 Harvard Medical School, Boston, MA USA

**Keywords:** Residency, Curriculum, Clinical competences, Education, Standards, Anesthesia, Competency based education

## Abstract

**Background:**

Competency-based medical education (CBME) has revolutionized approaches to training by making expectations more concrete, visible, and relevant for trainees. Designing, applying, and updating CBME requirements challenges residency programs, which must address many aspects of training simultaneously. This challenge also exists for educational regulatory bodies in creating and adjusting national competencies to standardize training expectations. We propose that an international approach for mapping residency training requirements may provide a baseline for assessing commonalities and differences. This approach allows us to take our first steps towards creating international competency goals to enhance sharing of best practices in education and clinical work.

**Methods:**

We chose anesthesiology residency training as our example discipline. Using two rounds of content analysis, we qualitatively compared published anesthesiology residency competencies for the European Union (The European Training Requirement), United States (ACGME Milestones), and Canada (CanMEDS Competence By Design), focusing on similarities and differences in representation (round one) and emphasis (round two) to generate hypotheses on practical solutions regarding international educational standards.

**Results:**

We mapped the similarities and discrepancies between the three repositories. Round one revealed that 93% of competencies were common between the three repositories. Major differences between European Training Requirement, US Milestones, and Competence by Design competencies involved critical emergency medicine. Round two showed that over 30% of competencies were emphasized equally, with notable exceptions that European Training Requirement emphasized Anaesthesia Non-Technical Skills, Competence by Design highlighted more granular competencies within specific anesthesiology situations, and US Milestones emphasized professionalism and behavioral practices.

**Conclusions:**

This qualitative comparison has identified commonalities and differences in anesthesiology training which may facilitate sharing broader perspectives on diverse high-quality educational, clinical, and research practices to enhance innovative approaches. Determining these overlaps in residency training can prompt international educational societies responsible for creating competencies to collaborate to design future training programs. This approach may be considered as a feasible method to build an international core of residency competency requirements for other disciplines.

**Supplementary Information:**

The online version contains supplementary material available at 10.1186/s12909-021-03007-w.

## Background

Competency-based medical education (CBME) has revolutionized approaches to training by making expectations more concrete, visible, and relevant for trainees [1, 2]. Yet overseeing a residency training program requires program directors, faculty and support staff, and institutions to nimbly adapt curricula to the ever-changing criteria for clinical excellence and competency-based medical education [1, 3]. The volume of medical knowledge approximately doubles in size every few months [4], so CBME requirements may change frequently and demand regular updates to account for these advancements [5]. The work to updating residency requirements is often performed at a national level, to homogenize some aspects of training, but the burden on institutional education programs to translate these guidelines is heavy and investments by volunteer committees may be uneven. The result is incomplete or patchy national and international diffusion of evidence-based practices at the residency training level in any individual country [6].

By matching the large-scale challenge of adapting curriculum through global processes, we can optimize resource management since all countries need to incorporate advancements on the same medical topics. But to do this, we need to have sufficiently common requirements for our training. This ambitious view mandates that we start with map of the current requirements for training. Outside of cardiac arrest resuscitation guidelines from the International Liaison Committee on Resuscitation [7, 8], we have few examples of shared competency training goals that are created between countries. A few recent studies have compared international training structures [9, 10], but none has considered individual competencies.

With this study we aimed to compare different regions’ requirements to create a baseline map of existing anesthesia program requirements. This could be the starting point for creating a shared set of competencies for future internationally agreed-upon standards for anesthesia or other specialties’ training programs. We hope to demonstrate that differences may be mapped in a way that allows for economies of effort and crowdsourcing to accelerate innovative educational design and reduce time wasted reinventing curricula. We used anesthesiology residency as a feasibility test for this conceptual approach.

## Methods

This study conforms to standards for reporting qualitative research (SRQR) [11]. Due to the nature of the study, Institutional Review Board involvement was not required.

### Context

During fellowship training of one researcher (CB), three researchers (CB, RE, and RDM) met and began discussions on the similarities and differences in our respective residency programs in EU (CB), Canada (RE), and US (RDM). We developed a collaborative research approach to compare published formal requirements quantitatively and qualitatively within those programs.

### Sampling strategy

We first identified each region’s current governance setting standards for anesthesiology residency education, which included: the European Board and Section of Anesthesiology working under the auspices of the European Union of Medical Specialists (UEMS) [12]; the Accreditation Council for Graduate Medical Education (ACGME) for the United States; and the Royal College of Physicians and Surgeons of Canada. We reviewed available metrics for anesthesiology residency educational assessments published by these governing bodies, following White and Marsh’s recommendations for choosing text to analyze [13]. Sources for consideration included the European Training Requirement (ETR) in Anesthesiology [14], the ACGME Program Requirements and the ACGME Milestones for US [15], and the Anesthesiology Competencies within Competence by Design (CBD) for Canada [16]. As the goal was to assess and compare published competencies, the ETR, the ACGME Milestones, and the Canadian Anesthesiology Competencies were chosen for comparison because they were most uniform in meeting our predefined criteria.

### Repository descriptions

#### The European training requirement (ETR) [14]

ETR were produced by the European Committee on Education and Professional Development of the Section and Board of Anesthesiology. At the time of this writing, the ETR were intended as a shared repository for all countries in the EU training anesthesiology residents. The latest version was dated February 2018. The scope of ETR was to offer “a *comprehensive and robust overall training framework created by medical specialists and based on assembled EU-wide educational and training experience*.” [14] Among the ETR objectives were facilitating professional mobility between European countries and promoting safe and quality care. ETR were not mandatory in EU countries, although the European Board and Section of Anesthesiology supported their adoption [17]. Nevertheless, repositories and certifications in EU’s countries were based upon or generally approximate ETR, though there were still some heterogeneity between European countries’ programs [18, 19]. Objectives of the ETR were part of a global framework with four generic roles: clinical expert, professional leader, academic scholar and inspired humanitarian. ETR consist of objectives, organized across 16 headlines (Supplemental Digital Content 1), which themselves belonged to two domains of (i) general competencies and (ii) specific core competencies. Each objective includes goals regarding knowledge, clinical skills and specific attitudes. The clinical skills comprised 165 items and the aimed mastery follows four grades of recommendations: (A) observer level (has knowledge of, describes); (B) performs, manages, demonstrates under direct supervision; (C) performs, manages, demonstrates under distant supervision; and (D) performs, manages, demonstrates independently. Depending on complexity of the skills or items, residents were expected to achieve anywhere from B (e.g. “Management of nerve blocks in pain therapy” or “Management of organ donors in Intensive care and during organ retrieval”), C (e.g. “Performing anesthesiology for kidney transplantation” or “Double lumen tracheal intubation”) or D grades (e.g. “Management of severe peri-partum hemorrhage” and “Management of difficult and delayed extubation after airway interventions”), with concessions given for rarer events.

#### The United States ACGME milestones [15]

The ACGME Milestones, introduced in 2013 but applied to anesthesiology in 2015, attempted to expand upon the six Core Competencies defined by the ACGME and the American Board of Medical Specialties which were Professionalism, Patient Care, Medical Knowledge, Practice-Based Learning and Improvement, Interpersonal and Communication Skills, and Systems-based Practice (Headlines in Supplemental Digital Content 2) [20]. The Milestones also intended to formalize the observations expected of residents within each of these six Core Competencies, driving residencies to teach and be assessed by how successfully their trainees met Milestones. Each specialty training programs’ Milestones were developed by experts within their specialty and varied in number of Milestones per specialty. As of 2020, anesthesiology comprised 25 Milestones. Residents attained one of five levels of achievement within each Milestone with clear behavioral definitions and anchors [15].

#### The Canadian CBD anesthesiology competencies [Entrustable professional activities (EPAs)] [16]

When this study was undertaken, two documents described the standards of achievement that are expected of Canadian anesthesiology residents when they were conferred fellowship in the Royal College of Physicians and Surgeons of Canada [16, 21]. The EPA guide summarized which EPAs residents were expected to achieve during their residency training (Headlines in Supplemental Digital Content 3). EPAs were clinical tasks which residents could perform with minimal or no supervision (i.e., the task can be entrusted to them to complete); these were considered the minimal standard of achievement expected at each stage of residency training. The Anesthesiology Competencies document provided a comprehensive description of the specialty of anesthesiology in Canada, and described what residents should aspire to achieve over their residency program [21]. The Anesthesiology Competencies were categorized in accordance with the CanMEDS competencies (i.e. Medical Expert, Communicator, Collaborator, Leader, Health Advocate, Scholar, Professional). EPAs were assessed with a 5-point scale of entrustability.

### Qualitative approach and research paradigm

From April to December 2019, we (CB, RE, and RDM) applied content analysis methodology [13, 22] and considered each of the training requirement repositories as data sources individually situated within an important cultural context, a distinction highlighted by Ratner [23] and uniquely suited to qualitative comparison as it allowed interpreting these training competencies through a sociocultural lens (pragmatic paradigm) [24, 25]. As our goal was to define similarities and differences between training expectations, we defined that each individually-numbered competency within any given repository would be considered the unit of data for comparison, which would be compared with all other competencies within the other two repositories.

### Data collection methods, processing and analysis

The three researchers (CB, RE, and RDM) entered each of the ETR, US Milestones, and CBD EPAs into a Microsoft Excel spreadsheet (version 2019) to serve as each region’s repository of competencies. Each competency item was reviewed by the investigator representing that region and compared with the other regions’ competencies to determine congruence. English versions of published competencies were available and were used for comparisons; all authors were fluent in English. As cultural influences were critical for analysis, we together discussed interpretative nuances of our representative region’s requirements and considered how anesthesiologists may have functioned in their scopes of practice in each of EU, US, and Canada. To avoid bias, each round consisted of separate and independent investigator review, followed by comparing and merging results, with discrepancies resolved through discussion and consensus, determining intersubjectivity [26]. For the first round of analysis, we identified whether each competency item was represented in either of the other repositories. The ETR was chosen as the reference comparison here, given that it had the greatest number of competencies. The second round of analysis sought to determine the relative importance or emphasis of specific skill sets in each country’s repository, using each repository as a reference for the other two to ensure full consideration of all competencies. Competency items were identified as having equal or different levels of importance, or “emphasis” between repositories based on how they were presented within the repository (i.e., competency items which were singled out and treated in-depth as unique competencies were interpreted as more emphasized than ones which were only briefly mentioned). For first round analysis, we sought to include rather than exclude, such that if a competency did not explicitly state an action, yet it could reasonably be included within the scope of the competency, common representation was considered between competencies. However, we applied stricter definitions whether domains were emphasized equally, and cultural context was considered more heavily. We used descriptive statistics to quantitatively present congruency and emphasis of competencies between the repositories.

### Researcher characteristics and reflexivity

Three investigators were practicing anesthesiologists having completed training in their respective regions: CB in EU (France), RE in Canada, and RDM in the US. All had domain expertise in education, including educational fellowships and advanced degrees. All three had served on their residency program’s clinical competency committees (either past or current). Two researchers were current or former residency or fellowship program directors (RE, RDM), and all actively taught anesthesiology residents at the time of data analysis.

### Techniques to enhance trustworthiness

Rigor was maintained by investigator independence at each stage, coupled with demonstrating intersubjectivity through discussion. Consensus was achieved universally between researchers through these discussions, with minimal instances of disagreement. In addition, external expert reviewers were invited to critique our results prior to submission (see Acknowledgements), lending credibility to our process. Based on their feedback, minor changes and correction were performed.

## Results

### Round 1: the broad view of shared competencies

**Fig. 1:**
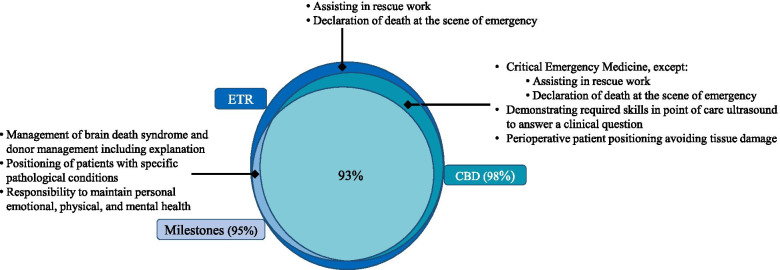
Venn diagram of common competencies for anesthesiology residency training for EU, US and Canada. The EU’s repository is the European Training Requirement (ETR), the US’ repository is the ACGME Milestones (Milestones), and the Canada’s repository is the Competence by Design (CBD). Incompletely matched competencies are described

Comparisons of ETR, US Milestones, and CBD EPAs competencies for anesthesiology resident training showed congruence of 93% (Fig. 1). All CBD EPAs and US Milestones’ competencies were present in the ETR. ETR competencies were represented in 98% of CBD EPAs and in 95% of US Milestones. CBD EPAs competencies were represented in 98% of US Milestones. US Milestones competencies were present in 96% of CBD EPAs. Table 1 summarizes the main results for the matching between ETR, US Milestones, and CBD EPAs’ competencies. Detailed results of the first round of comparisons of the three repositories’ competencies are presented in Appendix 1.

**Table 1:** Comparison of competencies for anaesthesiology residency training for EU, US, and Canada

ETR Domains’ headlines (number of items)	Number of items matching (%)		
	ETR with Milestones^a^	ETR with CBD^a^	ETR with Milestones with CBD^b^
Perioperative medicine, patient assessment and risk reduction (5)	5 (100)	5 (100)	5 (100)
General anaesthesia and sedation (25)	25 (100)	24 (96)	24 (96)
Airway management (4)	4 (100)	4 (100)	4 (100)
Regional anaesthesia (8)	8 (100)	7 (88)	7 (88)
Postoperative care and acute pain management (10)	10 (100)	10 (100)	10 (100)
Intensive care medicine (40)	40 (100)	40 (100)	40 (100)
Critical emergency medicine (CREM) (9)	0 (0)	7 (78)	0 (0)
Anaesthesia Non-Technical Skills (ANTS) (5)	5 (100)	5 (100)	5 (100)
Professionalism and ethics (8)	8 (100)	8 (100)	8 (100)
Patient safety and health economics (4)	4 (100)	4 (100)	4 (100)
Education, Self-directed Learning, Research (6)	6 (100)	6 (100)	6 (100)
Obstetric anaesthesiology (12)	12 (100)	12 (100)	12 (100)
Cardiothoracic anaesthesiology (9)	9 (100)	9 (100)	9 (100)
Neuroanaesthesiology (6)	6 (100)	6 (100)	6 (100)
Paediatric anaesthesiology (8)	8 (100)	8 (100)	8 (100)
Multidisciplinary chronic pain management (6)	6 (100)	6 (100)	6 (100)
**Total (165)**	**156 (95)**	**161 (98)**	**154 (93)**

^a^Comparison of CBD and US’ Milestones:

All but one (98%) CBD items were found in Milestones. The exception was C6: “*Demonstrating required skills in POCUS (point of care ultrasound) to answer a clinical question.*”

All but one (96%) of Milestones items were found in CBD. The exception was P5: “*Responsibility to maintain personal emotional, physical, and mental health.*”

^b^Comparison of ETR, CBD and US’ Milestones: see Appendix 1

The EU’s repository is the European Training Requirement (ETR), the US’ repository is the ACGME Milestones (Milestones), and the Canada’s repository is the Competence by Design (CBD). Incompletely matched competencies are described.

#### Differences between repositories

Most of the difference between ETR, US Milestones, and CBD EPAs’ competencies centered on critical emergency medicine (including pre-hospital and emergency medicine). This subject was completely absent from US Milestones. CBD EPAs’ competencies covered a similar field to the ETR for critical emergency medicine, except for “assisting in rescue work” and “declaration of death at the scene of emergency,” which were present only in the ETR. Skills in ultrasound and *Perioperative patient positioning avoiding tissue damage* were common for CBD EPAs and ETR but were absent from US Milestones. Three competencies were common to ETR and US Milestones and absent from CBD EPAs were: (1) *Management of brain death syndrome and donor management including explanation*; (2) *Positioning of patients with specific pathological conditions*; and (3) *Responsibility to maintain personal emotional, physical, and mental health*.

### Round 2: emphasis of certain competencies by ETR, milestones, and CBD

**Fig. 2:**
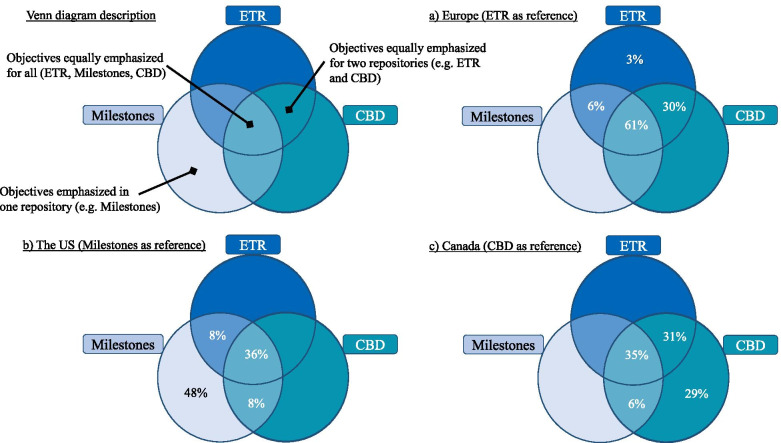
Venn diagrams of the competencies for anesthesiology residency training according to their relative emphasis in repositories for EU, US and Canada. The EU’s repository is the European Training Requirement (ETR), the US’ repository is the ACGME Milestones (Milestones), and the Canada’s repository is the Competence by Design (CBD). Expressions are in percentage of their repository according to perspectives from (**a**) ETR, (**b**) Milestones, and (**c**) CBD

Even if some competencies were cited in every repository, we did not determine all competencies had similar relative importance nor emphasis. Table 2 depicts which competencies were interpreted to have an equal expression among the three repositories, and which were emphasized equally by two or mainly by only one repository. This differential emphasis was demonstrated by comparing unique competencies such as “Promoting safety and well-being of staff” (ETR, 1.6) and “Responsibility to maintain personal, emotional, physical and mental health” (Milestones, P5) with how well-being was represented in CBD, which was dispersed within Core EPA #24, TTP #2, TTP #3, and TTP #5. Based on this deeper analysis, Venn diagrams (Fig. 2) depict the respective emphasis from each perspective (Europe, US, and Canada). More than 30 % of the competencies – whatever repository was taken as reference – had the same importance. ETR had only one (3%) competency that was specifically emphasized, Anaesthesia Non-Technical Skills (ANTS) [27]. Both CBD EPAs and US Milestones had more unique competencies emphasized. CBD EPAs focused on specific anesthesiology situations highlighting more granular competencies (e.g., point of care ultrasound skills, complex cases). Those competencies were included more generically for ETR and US Milestones, but without a high degree of detail. Professionalism and behavioral practices were emphasized by US Milestones (e.g., analysis of practice, education, communication).

**Table 2:** Common and emphasised anaesthesiology training competencies in EU, US and Canadian repositories

**Competencies equally represented for ETR, Milestones, CBD**		
Perioperative medicine, patient assessment, management plan, preparation, and risk reduction (Can.TTD1, Can.F1, Can.C1, Can.TTP1, E.1.1, US.PC1) Perioperative anaesthetic plan, management, conduct, and monitoring (Can.TTD2, Can.F2, E.1.2, US.PC2) Peri-procedural multimodal acute pain management, transfer of care, and postoperative orders (Can.TTD3, Can.C19, E.1.5, US.PC3, US.PC7) Regional anaesthesia (Can.C11, E.1.4, US.PC10) Assessing, diagnosing and managing critically ill patients in acute care settings (Can.F9, Can.F10, Can.C21, E.1.6, US.PC6) Airway management (Can.F4, Can.C4, E.1.3, US.PC8) ***– absence of extubation in US*** Assessing, diagnosing and managing chronic pain (Can.C20, E.2.5, US.PC7) Education, Self-directed Learning, Research (Can.TTP5, E.1.11, US.PBLI3) Honesty, integrity, and ethical behaviour (Can.C25, E.1.9, US.P2)		
**Competencies equally represented for CBD and ETR**	**Competencies equally represented for CBD and Milestones**	**Competencies equally represented for ETR and Milestones**
Obstetric anaesthesia and care; including providing labour analgesia, anaesthesia for caesarean, management of complications, management of high-risk parturient, and resuscitation of unstable parturient (Can.F12, Can.F13, Can.C7, Can.C8, Can.TTP4, E.2.1) Paediatric anaesthesia; including providing perioperative anaesthetic management, management of common complications (Can.F14, Can.F15, Can.F16, Can.C10, E.2.4) Providing anaesthetic management for patients undergoing procedures outside the usual environment of the operating room (Can.C13, E.1.2) Providing perioperative management for patients requiring shared airway procedures (Can.C14, E.1.2) Providing perioperative anaesthetic management for patients undergoing intracranial procedures (Can.C17, E.2.3) Providing perioperative anaesthetic management for patients undergoing thoracic surgery (CanC.18, E.2.2) Resuscitation for unstable patients, outside of the operating room or PACU (Can.C22, E.1.7) Initiating and leading resuscitation for unstable patients in the perioperative period (Can.C5, E.1.6)	Anticipating, preventing, diagnosing and managing common or expected peri-anaesthetic complications (Can.F6, Can.F8, US.PC4) Communication with patients and families (Can.C23, US.ICS1)	Responsibility to patients, families, and society (E.1.9, US.P1) Patient Safety and Quality Improvement (E.1.10, US.SBP2)
**Competencies emphasized in CBD**	**Competencies emphasized in ETR**	**Competencies emphasized in Milestones**
Managing and coordinating patient positioning during anaesthesia care and preventing and recognizing related complications (Can.F5) Assessing the indications for transfusion of blood products and managing side effects and complications (Can.F7) Providing anaesthetic management for patients with defined critical illness (Can.C3) Assessing, investigating, optimizing and formulating anaesthetic plans for more complex paediatric cases (Can.C9) Diagnosing and providing management for patients with complications of regional anaesthesia (Can.C12) Providing care for patients who have experienced a patient safety incident (Can.C24) Performing the non-airway basic procedures of anaesthesiology (Can.F3) Assessing pregnant patients and providing routine obstetric care or initial medical management for acute medical, surgical or obstetric conditions (Can.F11) Providing anaesthetic management for patients with defined critical illness (Can.C2) Demonstrating required skills in POCUS (point of care ultrasound) to answer a clinical question. (Can.C6) Providing perioperative management for patients requiring airway diagnostic and therapeutic procedures (Can.C15) Providing perioperative anaesthetic management for patients undergoing spinal procedures (Can.C16) Managing all aspects of anaesthesia care for a scheduled day list (Can.TTP2) Providing anaesthesia services for an on-call period (Can.TTP3)	Anaesthesia Non-Technical Skills (E.1.8)	Crisis management (US.PC5) Technical skills: Use and Interpretation of Monitoring and Equipment (US.PC9) Knowledge of biomedical, clinical, epidemiological, and social-behavioural sciences as outlined in the American Board of Anesthesiology Content Outline (US.MK1) Coordination of patient care within the health care system (US.SBP1) Team and leadership skills (US.ICS3) Incorporation of quality improvement and patient safety initiatives into personal practice (US.PBLI1) Analysis of practice to identify areas in need of improvement (US.PBLI2) Education of patient, families, students, residents, and other health professionals (US.PBLI4) Commitment to institution, department, and colleagues (US.P3) Receiving and giving feedback (US.P4) Responsibility to maintain personal emotional, physical, and mental health (US.P5) Communication with other professionals (US.ICS2)

*Can* Canada, *EPA* Entrustable Professional Activity, *TTD* Transition to Discipline EPA, *F* Foundation EPA, *C* Core EPA, *TTP* Transition to Practice EPA, *E* Europe, *US* United-States, *PC* Patient Care, *MK* Medical Knowledge, *SBP* Systems-based Practice, *PBLI* Practiced-based Learning and Improvement, *P* Professionalism, *ICS* Interpersonal and Communications Skills

Summarizes competencies common to the three repositories (EU, US and Canada), to two repositories (EU and Canada; US and Canada; or EU and US), or rather specific to one repository (EU, US or Canada). The EU repository is the European Training Requirement (ETR), the US repository is the ACGME Milestones (Milestones), and the Canadian repository is the Competence by Design (CBD). Expressions are in percentage of their repository according to perspectives from (a) ETR, (b) Milestones, and (c) CBD.

## Discussion

This comparison provides a glimpse into common training goals shared among Europe, the US and Canada, using anesthesiology as the example discipline. We identified a high overlap rate of educational objectives (93%). The core competencies appear generally consistent as shown in Table 2 (e.g., perioperative anesthetic plan, management, conduct, and monitoring; assessing, diagnosing and managing critically ill patients in acute care settings; airway management; and honesty, integrity, and ethical behavior, among others).

Differences on specific competencies emphasized are nuanced and likely have historical roots. For example, emergency medicine and prehospital medicine were initiated by anesthesiologists in some European countries, and anesthesiologists still play a large role in caring for prehospital patients despite emergency medicine internationally developing as a separate specialty [28–30]. Consideration is being given in the US for anesthesiologists to fill this gap via training in emergency medicine to care for critical patients [31]. A heavy societal focus on professionalism for US trainees likely has led to more defined behavioral guidelines regarding professional care, generating this unique emphasis in the US-based Milestones [32]. The large overlap identified in educational objectives with limited differences confirm that training appears very similar between regions and that anesthesiologists can work together, both at educational and professional levels. An important consideration for future work will be the differences in competencies required to practice in lower resourced or austere settings. The skills and knowledge required for clinicians practicing in wealthy, developed countries may not be the ones needed in developing countries. Understanding these differences among residency programs in developed nations highlighted in our study, and exploring the hypotheses stated above, should be topics for future investigation.

There are several reasons why this work has important potential. As countries strive to build better healthcare professionals through competency-based teaching and assessment, cross-cultural dialogue between international educational societies governing these competencies may enable effectively sharing best educational and clinical practices. For example, recently the Royal College of Anaesthetists in the UK adopted a curriculum update, entitled the 2021 Anaesthetics Curriculum, which included cultural values of diversity, inclusion, and respectful interactions between team members [33], and the Milestones were updated to include assessment of point-of-care ultrasound [34]. These should serve as exemplars for future updates to all repositories in anesthesiology. If and when this expands to countries with different cultural norms or different levels of technology and resources, other adaptations may need to be made. As regions like the EU, US, and Canada revise their CBME curricula and assessment tools, reviewing other countries’ successes and challenges in adopting changes will allow shared insights to be efficiently incorporated [33]. Thus, an educational global mindset may increase adaptation of new practices. In addition, best educational practices for adopting new techniques can offer faculty opportunities to refresh or gain new skills without reinventing educational approaches, saving expert time and organizational efforts.

Additionally, these requirements’ complete revision overburdens program directors, providing a substantial source of their burnout [35, 36]. By focusing on similarities, the workload of national and regional competency assessment efforts could be reduced, freeing program directors and educators to mentor trainees more effectively and adapt requirements for teaching using a regional lens. Engaging in such collaboration may facilitate other large-scale partnerships, such as subspecialty educational groups, to advance the educational sciences of teaching and learning in residency.

Many countries are in the process of developing more healthcare provider training programs or improving existing ones. By highlighting overlaps in competencies between CBME residency programs around the world, an international consensus of disciplines’ competencies may support creation of new training programs. Because best educational practices would be shared through international collaborations, this could facilitate training even in low-resource settings. Established training programs can also benefit from global innovations by identifying and closing their own educational gaps. Working towards a shared repository could be seen as investing time and energy in reciprocal learning, though everyone will gain from such a repository in distinct ways.

### Limitations

Our study has limitations that need highlighting. We focused on anesthesiology requirements in three wealthy, developed regions to illustrate our concept as the investigators are anesthesiologists. Generalizing to other specialties requires the existence of formally published competency repositories as a first step of many. Examining the process of creating competency repositories may serve as a preliminary step to unifying existing repositories, which could be aided by shared terminology and competency selection standards. We focused on a restricted area (EU, US, and Canada) as the investigators came from those areas (convenience sample). We would need to overcome difficulties to extend our analysis to other regions’ repositories. First, not every area has an easily accessible recent English version (or other shared language version) of their competencies, introducing language translation and interpretation challenges. Second, integrating and comparing more than three competencies would have been technically arduous. Nevertheless, we offer a robust methodology in comparing CBME training applicable across disciplines in healthcare.

The qualitative characteristic of this study may have limited the objectivity of the comparisons. To reduce this, we have had extensive discussion on the analysis for each item among the three researchers and data are available for consultation (Appendix 1). We also had an external independent review of the results.

The US and Canada have wide national application of their competencies. In EU, despite intentions to harmonize anesthesiology training [19], ETR adoption in its current form is less clear [18], and active complementary national ones coexist (e.g., UK or Denmark) [37, 38]. It is unclear how ETR have influenced the development of other EU national programs, as differences currently exist between length of training in EU countries, which may also lead to differential training, assessment, and regulation within the EU [18]. Gaps between these programs are not defined and only conjectural, as interpretation of competencies can vary by different program directors, specifically in EU where ETR application may differ from country to country. Furthermore, a final limitation remains that despite significant overlap in competencies, uniting accreditation practices between these three regions is likely to encounter barriers related to political and societal considerations.

### Perspectives and future directions

With now-defined competencies of CBME anesthesiology training programs for EU, US, and Canada, we call upon educational leaders from international societies to further develop competency standards. Basic technical skills (e.g. epidural, central line placement) and common competencies from Table 2 “Competencies equally represented for ETR, Milestones, CBD” (e.g. regional anesthesia, airway management) should benefit from developing common training programs which can become international standards. Future work should focus on determining the most effective teaching and assessment methodologies for achieving these competencies. Another exciting area of further development is using shared tools for competency assessment for licensure and certification, enabling anesthesiologists to temporarily practice in other countries during disasters.

## Conclusions

With this qualitative comparison, we were able to make a map discerning a baseline of similar competencies between published anesthesiology residency training competencies among EU, US, and Canada. Our approach also highlighted unique regional differences which appeared to be based on importance and approach rather than on fundamental content. Together, these serve as learning opportunities to explore. With over 90% overlap, the anesthesiology regional requirements we compared have enough in common to serve as a springboard to develop a common core of residency requirements in anesthesiology. This conceptual approach is demonstrated to be feasible and may be applied to determine a baseline from which to build an international core of residency competencies required by other disciplines.

## Supplementary Information

**Additional file 1: Appendix**.

**Additional file 2.** European Training Requirement Headlines (EU).

**Additional file 3.** ACGME Milestones headlines (US).

**Additional file 4.** Competence by Design headlines (Canada).

## Data Availability

All data generated or analyzed during this study are included in this published article [and its supplementary information files].

## Abbreviations

ACGME: Accreditation Council for Graduate Medical Education
CBD: Competence by Design
CBME: Competency-based medical education
EPA: Entrustable Professional Activity
ETR: European Training Requirement
EU: European Union
UEMS: European Union of Medical Specialists
US: United States
