# Supplementary material for: First steps towards international competency goals for residency training: a qualitative comparison of 3 regional standards in anesthesiology
Source: BMC Med Educ. 2021 Nov 10;21:569. doi: 10.1186/s12909-021-03007-w (PMC8582177; doi:10.1186/s12909-021-03007-w)
Supplement: Supplementary file 1 — Additional file 1: Appendix. [file 12909_2021_3007_MOESM1_ESM.docx]

**Appendix 1:** Detailed matching of competencies for anesthesiology residency training in EU [European Training Requirement (ETR)], US [ACGME Milestones (Milestones)], and Canada [Competence by Design (CBD)] using the descriptive items from the European Training Requirement as reference (16 headlines). Items for CBD are presented as Entrustable Professional Activities (EPA) distributed in four categories: Transition To Discipline (TTD) EPA, Foundation (F) EPA, Core (C) EPA, and Transition To practice (TTP) EPA. Items for Milestones are presented in six categories: Patient Care (PC), Medical Knowledge (MK), System-Based Practice (SBP), Practice-Based Learning and Improvement (PBLI), Professionalism (P), and Interpersonal and Communications Skills (ICS). Light grey color is used when the items are common to the three repositories. Medium grey color is used when the items are common to two of the repositories. Dark grey is used when the item is only in one repository. NA stands for Not Applicable when the item is not included in a repository.

|  | **ETR** | **Milestones** | **CBD** |
| --- | --- | --- | --- |
| **1.1 Perioperative medicine, patient assessment and risk reduction** |  |  |  |
| Patient assessment based on history and physical examination, use of appropriate examinations and laboratory tests in patients of all age groups with and without reduced functional cardiorespiratory capacity undergoing major and minor surgical routine and emergency interventions | 1.1 | PC1, PC8 | TTD1, F1, F2, C1, C15, TTP1, TTP2, TTP3 |
| Evaluation of the scores, including risks and ASA physical status | 1.1 | PC1, PC8 | TTD1, F1, F2, C1, C15, TTP1, TTP2, TTP3 |
| Evaluation of the airway | 1.1 | PC1, PC8 | TTD1, F1, F2, F4, C1, C15, TTP1, TTP2, TTP3 |
| Interpretation, considering the value and limitation of preoperative tests and monitoring | 1.1 | PC1, PC9, PC10 | TTD1, F2, F7, C1, C6, C15, TTP1, TTP2, TTP3 |
| Interdisciplinary patient optimization and risk reduction, including preoperative anaemia correction, cardiopulmonary treatment | 1.1 | PC1, ICS2, ICS3 | F2, C1, C15, TTP1, TTP2, TTP3 |
|  |  |  |  |
| **1.2 General anaesthesia and sedation** |  |  |  |
| Providing safe inhalation and intravenous induction, maintenance of, and emergence from general anaesthesia, including the choice of drugs, airway management, ventilation technique and intraoperative adverse event management | 1.2 | PC2 | F2, C2, C15, TTP2, TTP3 |
| Defibrillation, cardioversion | 1.2 | PC4, PC5 | C5, C15 |
| Aseptic techniques for invasive procedures including peripheral and central (ultrasound guided) venous access, intraosseous access, arterial catheterization, arterial blood gas collection, urinary catheterization, chest drain insertion | 1.2 | PC9 | F3, C2, C5, C15, TTP2, TTP3 |
| Gastrointestinal tube insertion | 1.2 | PC2 | C2 |
| Blood salvage (US: perform independently vs administer product processed by licensed individual) | 1.2 | PC2 | F7, C2, C5, C15, TTP2, TTP3 |
| Blood transfusion | 1.2 | PC2 | F7, C2, C5, C15, TTP2, TTP3 |
| Preparation of the workplace according relevant checklists and environmental safety measures | 1.2 | PC2 | TTD2, F2, C2, C15, TTP2, TTP3 |
| Use of medical and technical equipment appropriately, including neuromuscular blockade monitoring, volume monitoring, echocardiography | 1.2 | PC2, PC9 | F2, C2, C5, TTP2, TTP3 |
| Trouble-shooting basic technical malfunctions of monitors and machines | 1.2 | PC2, PC9 | F2, F6, C2, TTP2, TTP3 |
| Use of relevant checklists and guidelines | 1.2 | PC2 | TTD2, F2, C2, C15, TTP2, TTP3 |
| Monitoring nerve function during brain and spine surgery | 1.2 | PC2, PC9 | C16, TTP2, TTP3 |
| Perioperative patient positioning avoiding tissue damage | 1.2 | NA | F5, C2, C16, TTP2, TTP3 |
| Maintenance of homeostasis of organ systems throughout different surgical procedures in patients with and without pre-existing diseases | 1.2 | PC2 | F2, C2, C5, C15, TTP2, TTP3 |
| Diagnosis and management of intraoperative critical incidents | 1.2 | PC2, PC4, PC5, PC8 | F6, F7, C2, C5, C15, TTP2, TTP3 |
| Performing anaesthesia for laser airway surgery and interventions with a shared airway | 1.2 | PC2, PC8 | C4, C14, TTP2, TTP3 |
| Performing anaesthesia for fast track surgery and enhanced recovery after surgery | 1.2 | PC2 | F2, C2, TTP2, TTP3 |
| Performing anaesthesia in ICU patients | 1.2 | PC2, PC6 | C2, C5, TTP2, TTP3 |
| Performing sedation for invasive procedures | 1.2 | PC2 | C13, C14, TTP2, TTP3 |
| Performing anaesthesia and sedation outside the OR, taking into account organization of the site, type of procedures and patients | 1.2 | PC2 | C13, TTP2, TTP3 |
| Management of patient transport to and from remote locations | 1.2 | PC6 | C13, TTP2, TTP3 |
| Application of principles of safety during X-ray, MRI | 1.2 | SBP2 | C2, C13, TTP2, TTP3 |
| Application of discharge criteria for ambulatory anaesthesia | 1.2 | PC3 | TTP2, TTP3 |
| Consideration of ethical and medico-legal aspects | 1.2 | P2, ICS1 | TTD1, F1, F2, F6, C2, C5, TTP2, TTP3 |
| Initial surgical intervention in burn trauma and traumatic injury of the upper airway | 1.2 | PC2, PC6, PC9 | C2, C5, TTP2, TTP3 |
| Management of brain death syndrome and donor management including explanation | 1.2 | PC2, PC6 | NA |
|  |  |  |  |
| **1.3 Airway management** |  |  |  |
| Rapid sequence induction | 1.3 | PC2, PC8 | C2, C4, TTP2, TTP3 |
| Establishment and maintenance of an adequate airway in patients with anticipated and unanticipated difficult airway including patients with airway trauma and including the use of different devices and techniques according to existing algorithms | 1.3 | PC4, PC5, PC8 | C2, C4, C5, TTP2, TTP3 |
| Cricothyroidectomy (e.g. in medical simulation training) | 1.3 | PC4, PC5, PC8 | C4, C5 |
| Management of difficult and delayed extubation after airway interventions | 1.3 | PC4, PC8 | C2, C4, C5, TTP2, TTP3 |
|  |  |  |  |
| **1.4 Regional anaesthesia** |  |  |  |
| Performing neuraxial blocks such as spinal (single shot), thoracic epidural and lumbar epidural (single shot and catheter technique) combined spinal-epidural, caudal block | 1.4 | PC10 | F3, C2, C11 |
| Performing peripheral nerve blocks of the upper extremity (single shot and catheter technique) such as interscalene, axillary blocks | 1.4 | PC10 | C11 |
| Performing peripheral nerve blocks of the lower extremity (single shot and catheter technique) such as femoral, obturator, sciatic blocks | 1.4 | PC10 | C11 |
| Performing nerve blocks of the torso such as paravertebral, intercostal blocks | 1.4 | PC10 | C11 |
| Providing safe regional anaesthesia, including choice of drugs, techniques, and monitoring | 1.4 | PC10 | C11 |
| Positioning of patients with specific pathological conditions | 1.4 | PC10 | NA |
| Management of nerve blocks in pain therapy | 1.4 | PC7, PC10 | C19 |
| Diagnosis and management of intraoperative critical incidents | 1.4 | PC4, PC5, PC10 | reC11, C12 |
|  |  |  |  |
| **1.5 Postoperative care and acute pain management** |  |  |  |
| Providing handover of a patient in PACU (appropriate summary of relevant clinical features of the patient’s care) | 1.5 | PC4 | TTD3, C2, TTP2, TTP3 |
| Providing postoperative standard monitoring, indicating and interpreting individualized testing (e.g. ischemia monitoring, X-ray) | 1.5 | PC4, PC9 | F8, C2, TTP2, TTP3 |
| Pain assessment in all patient groups | 1.5 | PC3, PC7 | F8, C2, C19, TTP2, TTP3 |
| Use of relevant checklists and guidelines | 1.5 | PC2, PC3, PC4, PBLI2 | TTD3, F9, C2, TTP2, TTP3 |
| Maintenance of homeostasis of organ systems after the impact of different surgical procedures and anaesthesia in patients with and without pre-existing diseases | 1.5 | PC4, PC9 | F8, F9, C2, TTP2, TTP3 |
| Diagnosis and management of postoperative critical incidents (beyond those listed in domain 1.1) and postoperative adverse events | 1.5 | PC3, PC4, PC5, PC8, PC10 | F5, F8, F9, C2, C5, C19, TTP2, TTP3 |
| Detection of, indication for, and interprofessional organization of re-operation | 1.5 | PC7 | F9, C2, C5, TTP2, TTP3 |
| Performing weaning from supportive therapy of vital functions | 1.5 | PC7 | F9, C2, TTP2, TTP3 |
| Application of discharge criteria and transfer criteria to ICU | 1.5 | PC7 | F9, C2, C5, TTP2, TTP3 |
| Application of multimodal and pre-emptive analgesia concepts | 1.5 | PC4, PC7 | F8, C2, TTP2, TTP3 |
|  |  |  |  |
| **1.6 Intensive care medicine** |  |  |  |
| Performing patient assessment and physical examination including repetitive testing e.g. of peristaltic sounds, respiratory sounds, capillary refill, temperature gradient) | 1.6 | PC1, PC6 | F9, F10, C21, TTP3 |
| Identification of signs of instability of the cervical spine | 1.6 | PC1, PC5, PC6 | F9, C5, C21, TTP3 |
| Performing sedation, general anaesthesia, multimodal analgesia | 1.6 | PC2, PC6 | TTP3 |
| Performing neuraxial and peripheral nerve blocks for analgesia | 1.6 | PC3, PC6, PC10 | C19, TTP3, |
| Performing airway management including intubation under emergency situations | 1.6 | PC5, PC6, PC8 | C21, TTP3 |
| Performing aseptic insertion of venous, central venous, arterial, intra-osseus cannulation, pleural drainage | 1.6 | PC6 | F3, C21, TTP3 |
| Gastrointestinal tube insertion, urinary catheterization | 1.6 | PC6 | C21, TTP3 |
| Disease assessment and disease management | 1.6 | PC6, PC8, PC9 | F10, C5, C21 |
| Applying EBM-based therapeutic interventions, care bundles, guidelines protocols, and organ support in single or multiple organ failure (MODS) | 1.6 | PC6, PBLI3 | F10, C21, TTP3 |
| Patient transportation inter- and intra-hospital | 1.6 | PC6 | C21, TTP3 |
| Applying damage control and systematic priority-based approach in severe trauma patients | 1.6 | PC6 | C5, C21, TTP3 |
| Applying transfer criteria to specialized centres e.g. the critically ill child | 1.6 | PC6 | C21, TTP3 |
| Applying neuroprotection in head trauma and spinal cord trauma patients | 1.6 | PC6 | C5, C21, TTP3 |
| Performing general anaesthesia for repeated surgical interventions in burn trauma patients | 1.6 | PC2 | C2, TTP3 |
| Applying triage and prioritization of patients | 1.6 | PC6 | C5, TTP3 |
| Applying scoring systems (e.g. sedation depth, pain severity, APACHE, SAPS, TISS) | 1.6 | PC6 | C21, TTP3 |
| Performing basic ultrasound techniques for ultrasound-guided central venous line placement; recognition of severely abnormal ventricular function; measurement of inferior vena cava diameter; recognition of large pericardial, pleural, or abdominal effusion; recognition of urinary retention | 1.6 | PC6, PC9 | C5, C6, C21, TTP3 |
| Indicating, interpretation, considering the value and limitation of tests and monitoring | 1.6 | PC1, PC6, PC9, PC10 | F10, C5, C6, C21, TTP3 |
| Differential diagnosis, liaising with interdisciplinary specialists to interpret complex data | 1.6 | PC6, ICS2, ICS3 | F10, C5, C21 |
| Indicating physio- and ergotherapy | 1.6 | PC6, SBP1 | C21 |
| Consideration of ethical and medico-legal aspects | 1.6 | PC6 | F10, C21 |
| Performing regular visit rounds, ensuring continuity of care | 1.6 | PC6 | F10, C21 |
| Applying discharge criteria | 1.6 | PC6 | F10, C21 |
| Applying criteria for management change from curative to palliative care | 1.6 | PC6 | C21 |
| Providing handover of a patient to the ward (appropriate summary of relevant clinical features of the patient’s care) | 1.6 | PC6 | F10, C21 |
| Accurate record keeping | 1.6 | PC6, P1 | F10, C21 |
| Performing brain stem testing | 1.6 | PC6, PC9 | C21 |
| Management of organ donors in Intensive care and during organ retrieval | 1.6 | PC6 | C21 |
| Performing anaesthesia for kidney transplantation | 1.6 | PC2 | C2 |
| Performing immediate postoperative care of a kidney transplant patient | 1.6 | PC4, PC6 | C21 |
| has been exposed to the skills required to discuss with relatives about end of life issues, brain death and organ donation | 1.6 | PC6 | C21, C23 |
| *Effectively communicate with patients, treat patients with respect of basic ethical principles such as autonomy, privacy, dignity, confidentiality, including discussing end of life decisions* | 1.6 | PC1, PC6, SBP1, PBLI4, P1, P2, ICS1 | F10, C21, C23 |
| *Establishing effective interaction with patients, including patients with impaired capacity of discernment and consent and their relatives* | 1.6 | PC1, PC6, SBP1, PBLI4, P1, P2, ICS1 | F10, C21, C23 |
| *Effectively communicate with patients with language barriers* | 1.6 | PC1, PC6, SBP1, PBLI4, P1, P2, ICS1 | F10, C21, C23 |
| *Effectively communicate with other health care providers* | 1.6 | PC1, PC5, PC6, SBP1, PBLI1, PBLI4, P1, P2, P3, P4, ICS1, ICS2, ICS3 | F10, C21, C23 |
| *Team work together with other health care professionals to ensure smooth patient care and safety* | 1.6 | PC1, PC5, PC6, SBP1, PBLI1, PBLI4, P1, P2, P3, P4, ICS1, ICS2, ICS3 | F10, C5, C21 |
| *Vigilance and situational awareness* | 1.6 | PC5, PC6, SBP1, SBP2, PBLI1, P1, ICS2 | F10, C5, C21 |
| *Respecting legal constraints* | 1.6 | PC6, SBP1, P1, P2, ICS1 | F10, C21 |
| *Promoting safety and well-being of staff* | 1.6 | PC6, SBP1, SBP2, PBLI1, P1, P3, P5 | C21 |
| *Promoting infection control measures* | 1.6 | PC6, SBP2, PBLI1 | C21 |
|  |  |  |  |
| **1.7 Critical emergency medicine (CREM)** |  |  |  |
| Applying skills from domains 1.1 to 1.5 in pre-hospital critical emergency scenarios | 1.7 | NA | F9, C22, TTP3 |
| Management of life-threatening medical and surgical emergency conditions | 1.7 | NA | F9, C5, C21, C22, TTP3 |
| Applying resuscitation algorithms and trauma guidelines | 1.7 | NA | F9, C5, C21, C22, TTP3 |
| Assisting in rescue work | 1.7 | NA | NA |
| Performing emergency medicine in the interdisciplinary team of an emergency room | 1.7 | NA | C5, C21, C22, TTP3 |
| Performing intra-hospital resuscitation in the interdisciplinary cardiac arrest team | 1.7 | NA | C5, C21, C22, TTP3 |
| Performing echocardiography for fast differential diagnosis (FAST approach) | 1.7 | NA | F9, C5, C6, C21, C22, TTP3 |
| Supporting the complex organization of health care in cases of mass accidents and disasters | 1.7 | NA | TTP3 |
| Declaration of death at the scene of emergency | 1.7 | NA | NA |
|  |  |  |  |
| **1.8 Anaesthesia Non-Technical Skills (ANTS)** |  |  |  |
| Task management | 1.8 | PC1, PC5, PC6, SBP1, SBP2, PBLI1, P1, P3, ICS2 | F6, C2, C5, TTP1, TTP2, TTP3 |
| Team working | 1.8 | PC5, PC6, SBP1, SBP2, PBLI1, PBLI4, P1, P3, ICS2, ICS3 | C2, C5, TTP1, TTP2, TTP3 |
| Situation Awareness | 1.8 | PC5, PC6, SBP1, SBP2, PBLI1, P1, P3, ICS2 | F6, C2, C5, TTP1, TTP3 |
| Decision making | 1.8 | PC5, PC6, SBP1, SBP2, PBLI1, PBLI4, P1, P3, ICS2 | C2, C5, TTP1, TTP2, TTP3 |
| Leadership | 1.8 | PC5, PC6, SBP1, SBP2, PBLI1, P1, P3, ICS2, ICS3 | C2, C5, TTP1, TTP2, TTP3 |
|  |  |  |  |
| **1.9 Professionalism and ethics** |  |  |  |
| Applying principles of medical ethics to problem solving | 1.9 | PC1, PC2, PC6, SBP1, P1, P2, P3, ICS1 | C2, C5, C23, C25, TTP2, TTP3 |
| Attaining attributes in the 4 roles of a specialist in anaesthesiology: medical expert, leader; scholar; professional | 1.9 | PC5, PC6, SBP1, PBLI4, P1, P2, P3, ICS2, ICS3 | F11, C2, C5, TTP2, TTP3 |
| Applying the principles of evidence-based medicine to clinical practice | 1.9 | PC6, MK1, PBLI3 | C2, C5, TTP2, TTP3 |
| Use of information technology in order to optimize clinical care, conducting literature searches | 1.9 | PC6, MK1, PBLI3 | TTP2, TTP3 |
| Basic appraising journal articles including the interpretation of study design, statistics, results, and conclusions | 1.9 | PC6, MK1, PBLI3 | TTP2, TTP3 |
| Awareness and management according to medico-legal obligations related to medical practice | 1.9 | PC1, PC2, PC4, PC6, P1, P2 | C24, TTP2, TTP3 |
| Commitment to the main ethical principles and professional values, such as altruism, fidelity, social justice, honour, integrity, and accountability | 1.9 | PC1, PC2, PC6, SBP1, PBLI1, P1, P2, P3, ICS1, ICS2, ICS3 | F11, C5, C24, C25, TTP2, TTP3 |
| Commitment to the rights of patients to autonomy, confidentiality, informed consent, comprehension of the risks of medical techniques (patient-centeredness) irrespectively of race, culture, gender, sexual orientation, and socio-economic status | 1.9 | PC1, PC2, PC6, SBP1, P1, P2, P3, ICS1, ICS2 | F11, C5, C24, TTP2, TTP3 |
|  |  |  |  |
| **1.10 Patient safety and health economics** |  |  |  |
| Application of standards of quality of care and patient safety in daily practice including anaesthesia in remote locations | 1.10 | PC2, SBP2, PBLI1, PBLI2, ICS1 | F6, C2, C5, TTP2, TTP3 |
| Use of checklists and guidelines | 1.10 | PC2, PC5, SBP2, PBLI1, ICS1 | C2, C5, TTP2, TTP3 |
| Providing data for both local and national data systems | 1.10 | SBP2 | C2, C5, TTP2, TTP3 |
| Considering cost-effectiveness | 1.10 | SBP2, P1 | C2, TTP2, TTP3 |
|  |  |  |  |
| **1.11 Education, Self-directed Learning, Research** |  |  |  |
| Conducting and appraising literature searches | 1.11 | MK1, PBLI3 | TTP5 |
| Appraising journal articles including the application of statistics | 1.11 | MK1, PBLI3 | TTP5 |
| *Applying the principles of evidence-based medicine to clinical practice (identic to 1.9)* | 1.11 | MK1, PBLI3 | C2, TTP2, TTP3, TTP5 |
| Carrying out oral presentations and professional communication | 1.11 | SBP2, PBLI4, ICS2, ISC3 | TTP5 |
| Presenting quality assurance exercises or projects | 1.11 | SBP2, PBLI1, PBLI2, ICS1 | F6, TTP5 |
| Developing facilitation skills, such as tutoring in small-group learning and conducting small group meetings | 1.11 | SBP2, ICS1, ICS2, ICS3 | TTP2, TTP3, TTP5 |
|  |  |  |  |
| **2.1 Obstetric anaesthesiology** |  |  |  |
| Applying skills from domains 1.1 to 1.4 in parturients including | 2.1 | PC1, PC2, PC4, PC5, PC8 | F11, F13, C7, TTP4 |
| Positioning of parturients | 2.1 | PC2 | C7, TTP4 |
| Performing anaesthesia for delivery | 2.1 | PC2 | F12, C7, TTP4 |
| Performing spinal anaesthesia (single shot), combined spinal-epidural anaesthesia and lumbar epidural anaesthesia (single shot and catheter technique) for caesarean section | 2.1 | PC10 | F13, C7, TTP4 |
| Management of pain in pregnancy and labour | 2.1 | PC3 | F12, C7, TTP4 |
| Performing lumbar epidural catheter placement for labour analgesia | 2.1 | PC10 | F12, C7, TTP4 |
| Management of severe peri-partum haemorrhage | 2.1 | PC5 | C7, C8, TTP4 |
| Initial management of high-risk parturients and application of transfer criteria to higher level hospitals | 2.1 | PC5 | C7, TTP4 |
| Performing anaesthesia in pregnant and breastfeeding women | 2.1 | PC2 | C2, TTP4 |
| Performing anaesthesia and analgesia in assisted reproductive technologies and intrauterine surgery | 2.1 | PC2 | C7 |
| Applying skills from domains 1.7 to 1.9 in parturients | 2.1 | PC1, PC2, PC6 | F10, C8, TTP4 |
| Basic and advanced life support, including resuscitation of the newborn (+ Accurate record keeping) | 2.1 | PC5, P1 | F10, F12, F13, C8, TTP4 |
|  |  |  |  |
| **2.2 Cardiothoracic anaesthesiology** |  |  |  |
| Specific respiratory evaluation with regards to planned surgery (assessment of operability) | 2.2 | PC1, PC8 | C18 |
| Performance of lung separation techniques (double lumen intubation, fiberoptic control and tube positioning) | 2.2 | PC2, PC8 | C18 |
| Patient positioning, particularly in the lateral decubitus position | 2.2 | PC2 | C18 |
| Using chest tube drainage systems and suction | 2.2 | PC2 | C18 |
| Basic skills in the management of anaesthesia and perioperative care for cardiac operations performed on-pump and off-pump | 2.2 | PC2 | C3 |
| Use of advanced haemodynamic monitoring | 2.2 | PC2, PC9 | C3, C6, C15 |
| Use of TEE for evaluation of size and function of left and right ventricle, all valves (stenosis, insufficiency, severity), diagnosis of pericardial fluid or tamponade, dilation or dissection of the aorta | 2.2 | PC9 | C3, C6, C15 |
|  |  |  |  |
| **2.3 Neuroanaesthesiology** |  |  |  |
| Specific evaluation with regards to planned surgery (assessment of operability) | 2.3 | PC1 | C17 |
| Patient positioning, particularly in the sitting position | 2.3 | PC2 | C17 |
| Management of specific complications including air embolism, intracranial hypertension | 2.3 | PC2, PC4, PC5 | C17 |
| Basic skills in the management of anaesthesia and perioperative care for intracranial operations, including induced hypotension, induced hypothermia | 2.3 | PC2 | C17 |
| Apply principles of neuroprotection | 2.3 | PC2, PC6 | C17 |
| Use and interpretation of advanced neuromonitoring (e.g. evoked potentials, cerebral oxygenation, blood flow, metabolism) | 2.3 | PC2, PC9 | C17 |
|  |  |  |  |
| **2.4 Paediatric anaesthesiology** |  |  |  |
| Applying skills from domains 1.1 to 1.4 in paediatric patients > 1 year of age | 2.4 | PC1, PC2 | F14, F16, C9 |
| Performing vascular access in young children < 1 year | 2.4 | PC2 | F14, C10 |
| Performing airway management in young children < 1 year | 2.4 | PC8 | C10 |
| Performing general anaesthesia in young children < 1 year | 2.4 | PC2 | C10 |
| Performing peripheral and neuraxial regional blocks including caudal anaesthesia in young children < 1 year | 2.4 | PC10 | C10 |
| Performing postoperative care, pain management, general intensive care in young children < 1 year | 2.4 | PC3, PC4 | F15, C10 |
| Performing cardiorespiratory resuscitation in children and neonates | 2.4 | PC5 | C10 |
| Recognizing patients that should be transferred to a higher competence facility and safely transfer them | 2.4 | PC4, PC6 | C10 |
|  |  |  |  |
| **2.5 Multidisciplinary chronic pain management** |  |  |  |
| Evaluation of patients with chronic pain: history, physical examination and requesting and interpretation of additional tests considering the bio-psycho-social model | 2.5 | PC1, PC3, PC7 | C20 |
| Applying pain scales and validated questionnaires | 2.5 | PC3, PC7 | C20 |
| Explaining treatment options and clinical goals | 2.5 | PC1, PC3, PC7 | C20 |
| Initial multimodal treatment of patients with chronic cancer and non-cancer pain | 2.5 | PC3, PC7 | C20 |
| Diagnosis and management of adverse effects of pain therapy | 2.5 | PC3, PC7 | C20 |
| Accurate record keeping (logbook), including treatments and procedures. Documentation of pain evolution | 2.5 | PC7, P1 | C20 |
