# Supplementary material for: First steps towards international competency goals for residency training: a qualitative comparison of 3 regional standards in anesthesiology
Source: BMC Med Educ. 2021 Nov 10;21:569. doi: 10.1186/s12909-021-03007-w (PMC8582177; doi:10.1186/s12909-021-03007-w)
Supplement: Supplementary file 3 — Additional file 3. ACGME Milestones headlines (US). [file 12909_2021_3007_MOESM3_ESM.docx]

# ACGME Milestones headlines (US)

| **Category** | **Description** |
| --- | --- |
| Patient Care 1 | Pre-anesthetic Patient Evaluation, Assessment, and Preparation |
| Patient Care 2 | Anesthetic Plan and Conduct |
| Patient Care 3 | Peri-procedural pain management |
| Patient Care 4 | Management of peri-anesthetic complications |
| Patient Care 5 | Crisis management |
| Patient Care 6 | Triage and management of the critically-ill patient in a non-operative setting |
| Patient Care 7 | Acute, chronic, and cancer-related pain consultation and management |
| Patient Care 8 | Airway management |
| Patient Care 9 | Use and Interpretation of Monitoring and Equipment |
| Patient Care 10 | Regional anesthesia |
| Medical Knowledge 1 | Knowledge of biomedical, clinical, epidemiological, and social-behavioral sciences as outlined in the American Board of Anesthesiology Content Outline |
| Systems-based Practice 1 | Coordination of patient care within the health care system |
| Systems-based Practice 2 | Patient Safety and Quality Improvement |
| Practiced-based Learning and Improvement 1 | Incorporation of quality improvement and patient safety initiatives into personal practice |
| Practiced-based Learning and Improvement 2 | Analysis of practice to identify areas in need of improvement |
| Practiced-based Learning and Improvement 3 | Self-directed learning |
| Practiced-based Learning and Improvement 4 | Education of patient, families, students, residents, and other health professionals |
| Professionalism 1 | Responsibility to patients, families, and society |
| Professionalism 2 | Honesty, integrity, and ethical behavior |
| Professionalism 3 | Commitment to institution, department, and colleagues |
| Professionalism 4 | Receiving and giving feedback |
| Professionalism 5 | Responsibility to maintain personal emotional, physical, and mental health |
| Interpersonal and Communications Skills 1 | Communication with patients and families |
| Interpersonal and Communications Skills 2 | Communication with other professionals |
| Interpersonal and Communications Skills 3 | Team and leadership skills |
