# Supplementary material for: First steps towards international competency goals for residency training: a qualitative comparison of 3 regional standards in anesthesiology
Source: BMC Med Educ. 2021 Nov 10;21:569. doi: 10.1186/s12909-021-03007-w (PMC8582177; doi:10.1186/s12909-021-03007-w)
Supplement: Supplementary file 4 — Additional file 4. Competence by Design headlines (Canada). [file 12909_2021_3007_MOESM4_ESM.docx]

# Competence by Design headlines (Canada)

| **Category** | **Description** |
| --- | --- |
| Transition to Discipline EPA #1 | Performing preoperative assessments for healthy adult patients who will be undergoing a non-complex scheduled surgical procedure |
| Transition to Discipline EPA #2 | Monitoring adult patients undergoing non-complex surgical procedures, under general or regional anesthesia |
| Transition to Discipline EPA #3 | Performing the postoperative transfer of care of healthy adult patients following a non-complex surgical procedure, including postoperative orders |
| Foundation EPA #1 | Using the anesthetic assessment to generate the anesthetic considerations and management plan including postoperative disposition, and obtaining informed consent, for non-complex patients and non-complex surgery |
| Foundation EPA #2 | Providing perioperative anesthetic management for non-complex cases in adult patients |
| Foundation EPA #3 | Performing the non-airway basic procedures of Anesthesiology |
| Foundation EPA #4 | Identifying patients presenting with an anticipated difficult airway and preparing for management options |
| Foundation EPA #5 | Identifying patients presenting with an anticipated difficult airway and preparing for management options |
| Foundation EPA #6 | Anticipating, preventing and managing common or expected intraoperative events and physiologic changes during non-complex cases |
| Foundation EPA #7 | Assessing the indications for transfusion of blood products and managing side effects and complications |
| Foundation EPA #8 | Diagnosing and managing common issues in the post-anesthesia care unit (PACU), or the surgical ward |
| Foundation EPA #9 | Initiating resuscitation and diagnosis of patients with life-threatening conditions in a time-appropriate manner |
| Foundation EPA #10 | Assessing, diagnosing and managing patients with common medical or surgical presentations in acute care settings, and advancing their care plans |
| Foundation EPA #11 | Assessing pregnant patients and providing routine obstetric care or initial medical management for acute medical, surgical or obstetric conditions |
| Foundation EPA #12 | Assessing and providing labour analgesia for healthy parturients with an uncomplicated pregnancy, including the management of common complications of labour analgesia |
| Foundation EPA #13 | Providing anesthesia for patients undergoing non-complex cesarean section |
| Foundation EPA #14 | Providing perioperative anesthetic management for non-complex cases in pediatric patients |
| Foundation EPA #15 | Managing pediatric patients with common postoperative complications in the post anesthesia care unit or ward |
| Foundation EPA #16 | Assessing and initiating management for pediatric patients with common medical conditions |
| Core EPA # 1 | Using the anesthetic assessment to generate the anesthetic considerations and management plan, including prioritization and optimization, for patients with complex medical issues or surgeries |
| Core EPA # 2 | Providing anesthetic management for patients with defined critical illness |
| Core EPA # 3 | Providing perioperative anesthetic management for patients with significant cardiac disease |
| Core EPA # 4 | Managing patients presenting with a difficult airway, including developing plans for extubation |
| Core EPA # 5 | Initiating and leading resuscitation for unstable patients in the perioperative period |
| Core EPA # 6 | Demonstrating required skills in POCUS (point of care ultrasound) to answer a clinical question |
| Core EPA # 7 | Providing peripartum anesthetic management for high-risk parturients |
| Core EPA # 8 | Initiating resuscitation and providing anesthetic management for unstable parturients |
| Core EPA # 9 | Assessing, investigating, optimizing and formulating anesthetic plans for more complex pediatric cases |
| Core EPA # 10 | Providing perioperative anesthetic management for pediatric patients with more complex cases |
| Core EPA # 11 | Providing perioperative anesthetic management incorporating a peripheral nerve block technique |
| Core EPA # 12 | Diagnosing and providing management for patients with complications of regional anesthesia |
| Core EPA # 13 | Providing anesthetic management for patients undergoing procedures outside the usual environment of the operating room |
| Core EPA # 14 | Providing perioperative management for patients requiring airway diagnostic and therapeutic procedures |
| Core EPA # 15 | Providing perioperative anesthetic management for patients undergoing vascular surgery |
| Core EPA # 16 | Providing perioperative anesthetic management for patients undergoing spinal procedures |
| Core EPA # 17 | Providing perioperative anesthetic management for patients undergoing intracranial procedures |
| Core EPA # 18 | Providing perioperative anesthetic management for patients undergoing thoracic surgery |
| Core EPA # 19 | Assessing and providing comprehensive multi-modal management for patients with complex acute pain |
| Core EPA # 20 | Assessing, diagnosing and formulating management options for patients with common chronic pain disorders |
| Core EPA # 21 | Providing comprehensive ongoing management of critically ill patients in an intensive care setting |
| Core EPA # 22 | Initiating and leading resuscitation for unstable patients, outside of the operating room or PACU |
| Core EPA # 23 | Managing goals of care discussions with patients and families, including perioperative care plans |
| Core EPA # 24 | Providing care for patients who have experienced a patient safety incident |
| Core EPA # 25 | Recognizing and managing ethical dilemmas that arise in the course of patient care |
| Transition to Practice EPA #1 | Managing all aspects of care for patients presenting to a preoperative clinic |
| Transition to Practice EPA #2 | Managing all aspects of anesthesia care for a scheduled day list |
| Transition to Practice EPA #3 | Providing anesthesia services for an on-call period |
| Transition to Practice EPA #4 | Managing all aspects of care for obstetrical anesthesia services |
| Transition to Practice EPA #5 | Developing an academic portfolio |
